# Supplementary material for: Combined Inhibition of TGF-β1-Induced EMT and PD-L1 Silencing Re-Sensitizes Hepatocellular Carcinoma to Sorafenib Treatment
Source: J Clin Med. 2021 Apr 27;10(9):1889. doi: 10.3390/jcm10091889 (PMC8123871; doi:10.3390/jcm10091889)
Supplement: Supplementary file 1 [file jcm-10-01889-s001.zip › jcm-1171724-supplementary.pdf]

Journal of
Clinical Medicine

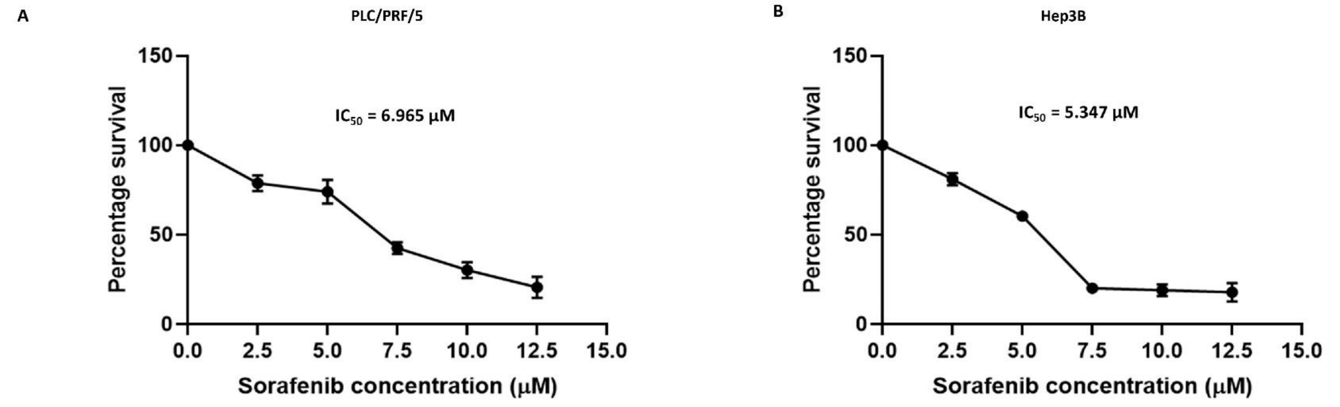

Figure S1. IC50 concentration of Sorafenib in HCC cells. Cell viability assay demonstrated percentage survival of HCC cells at various dose concentration of Sorafenib (μM). The GraphPad analysis provided IC50 values of Sorafenib as 6.965 μM and 5.347 μM in PLC/PRF/5 and Hep3B cells, respectively.

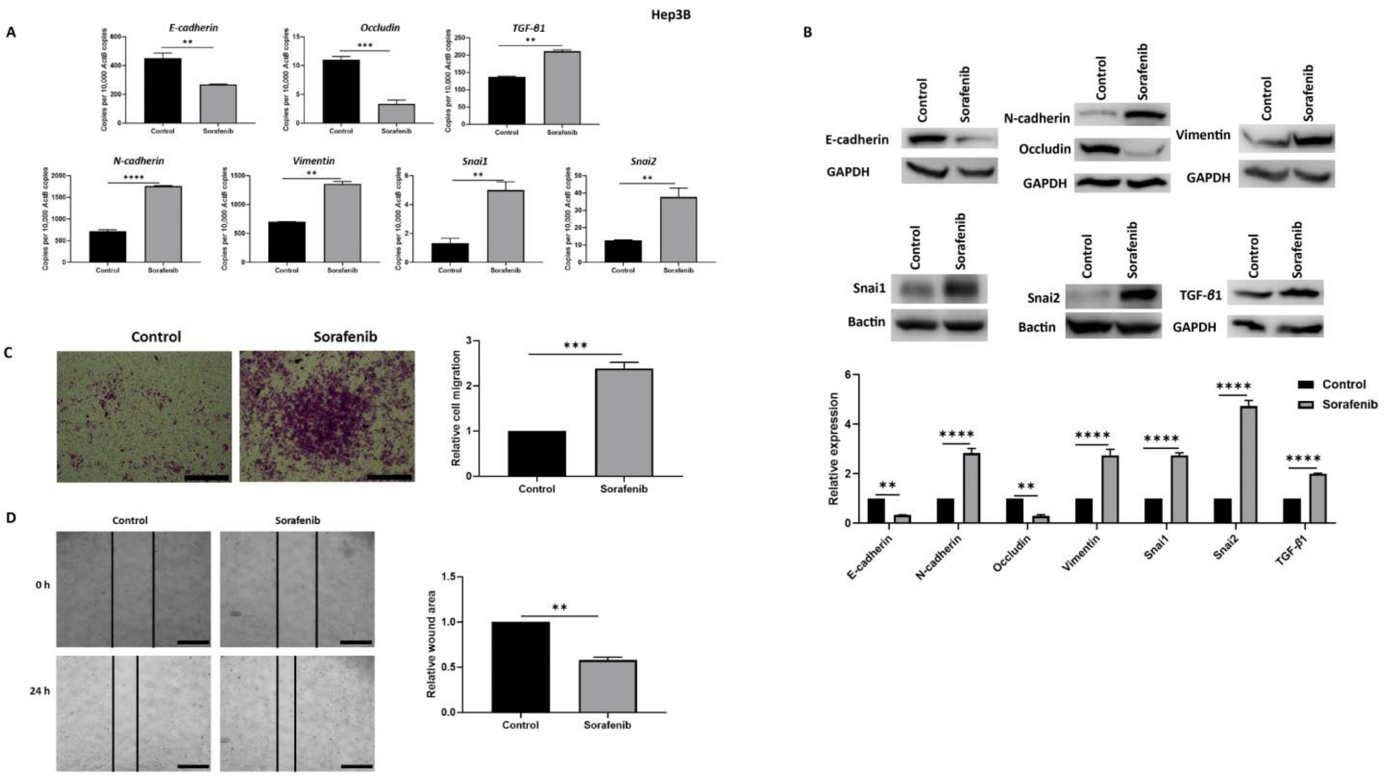

Figure S2. Sorafenib treatment activates EMT in Hep3B cells. (A) qRT-PCR revealed reduction in E-cadherin and Occludin expression and increase in TGF-β1, N-cadherin, Vimentin, Snai1 and Snai2 expression after treatment with 5.347 μM Sorafenib for 72 h. (B) Western blot analysis showed repression of E-cadherin and Occludin and induction of Vimentin, N-cadherin, Snai1, Snai2 and TGF-β1 upon treatment with Sorafenib. GAPDH and Bactin were used as loading controls. (C) The migratory capability of Hep3B cells was increased upon treatment with Sorafenib as revealed by transwell migration assay (scale bar = 500 μm). The number of motile cells was proportional to the absorbance of Crystal Violet staining. (D) Wound healing assay (scale bar = 500 μm) showed higher migratory capacity of Sorafenib treated Hep3B cells. Quantitative analysis of wound area after 24 h Sorafenib treatment relative to the starting wound area at 0 h. (n=3, ** $p<0.01$, *** $p<0.005$, ****$p<0.001$).

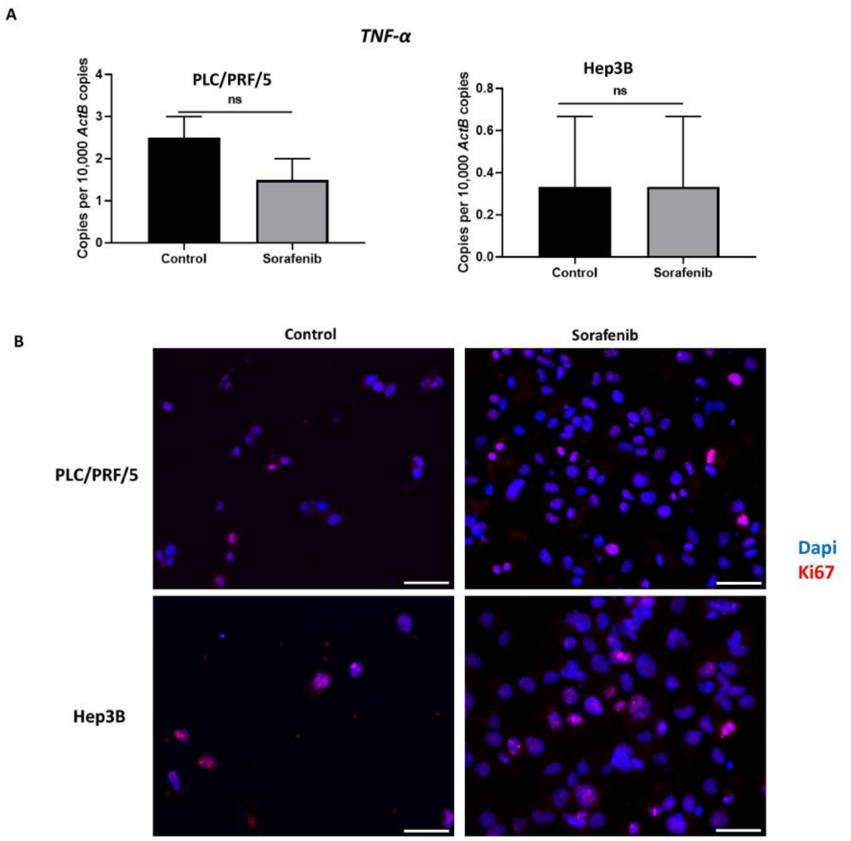

Figure S3. Sorafenib treatment in HCC cells. (A) qRT-PCR demonstrated no upregulation of TNF-α expression following treatment with Sorafenib in both PLC/PRF/5 and Hep3B cells. (B) Fluorescence microscopy revealed positive Ki67 staining for cells migrated through transwell membrane following Sorafenib treatment (scale bar = 200 μm).

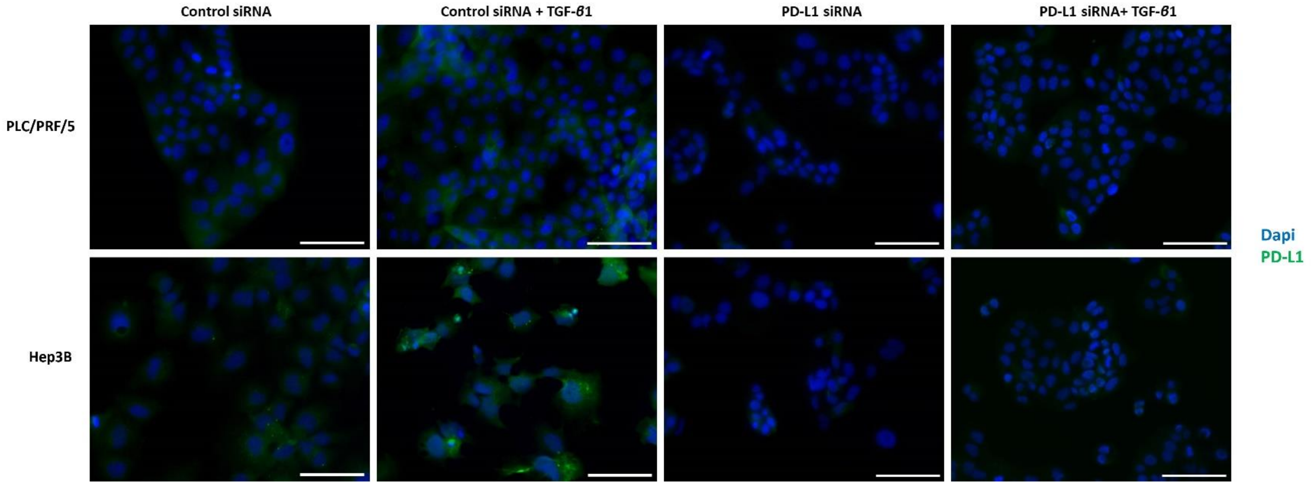

Figure S4. Silencing of PD-L1 in HCC cells. Fluorescence microscopy demonstrated reduced expression of PD-L1 in TGF-β1 stimulated cells following PD-L1 knockdown assay in PLC/PRF/5 and Hep3B cells.

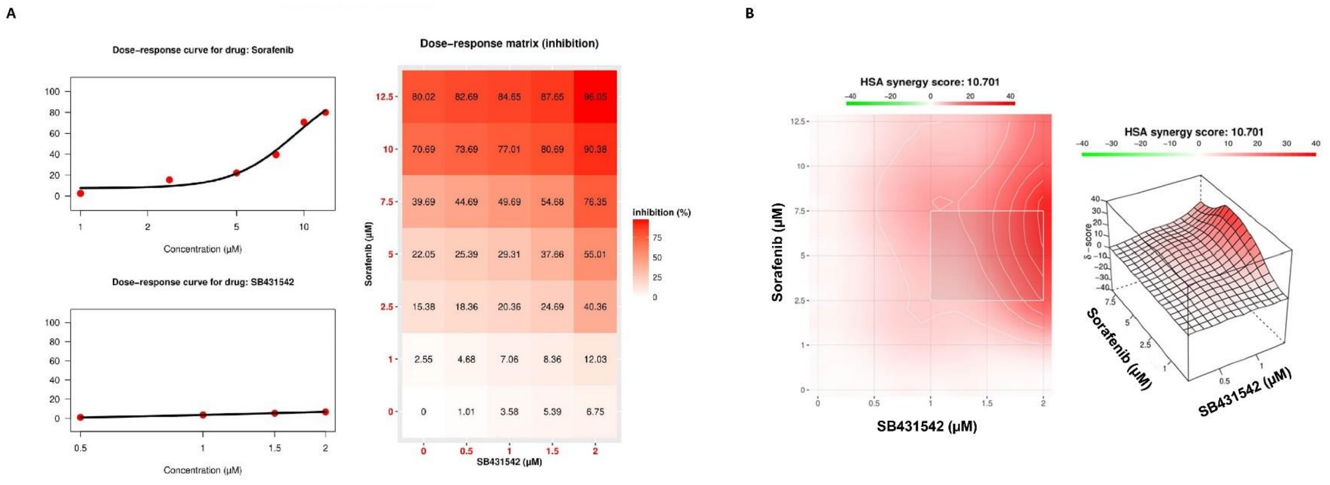

Figure S5. Drug combination of Sorafenib and SB431542 shows synergistic inhibition effect. (A) Dose-response matrix for Sorafenib and SB431542 combination. (B) HSA synergy score of drug combination for Sorafenib and SB431542.

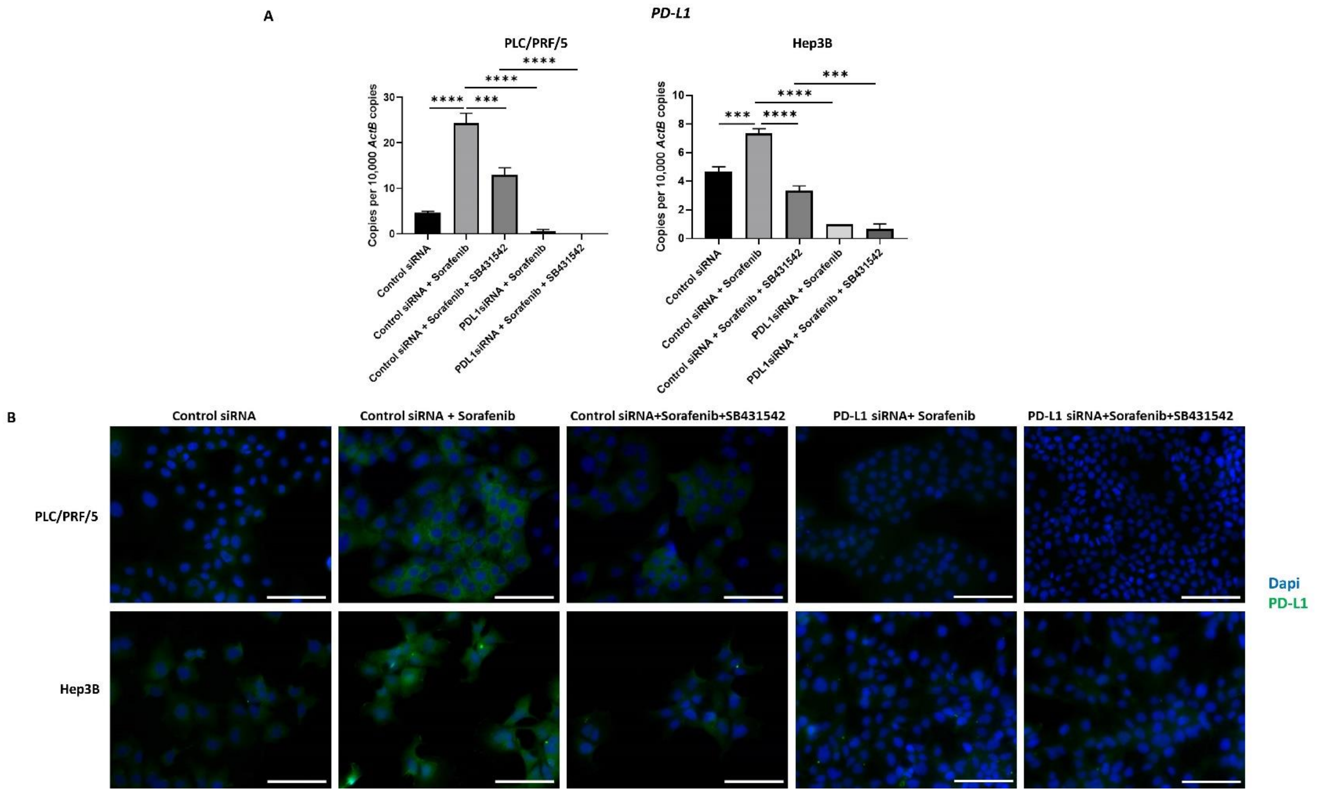

Figure S6. Combination treatment of Sorafenib with PD-L1 knockdown and SB431542 reduces expression of PD-L1 in HCC cells. Combination treatment of Sorafenib with PD-L1 knockdown and SB431542 significantly reduced expression of PD-L1 in both PLC/PRF/5 and Hep3B cells as demonstrated by (A) qRT-PCR and (B) fluorescence microscopy (scale bar = 200 μm). (n=3, *** $p<0.05$, ****$p<0.001$).
